# Supplementary material for: Anti-Adipogenic Effect of Theabrownin Is Mediated by Bile Acid Alternative Synthesis via Gut Microbiota Remodeling
Source: Metabolites. 2020 Nov 23;10(11):475. doi: 10.3390/metabo10110475 (PMC7700314; doi:10.3390/metabo10110475)
Supplement: Supplementary file 1 [file metabolites-10-00475-s001.pdf]

Anti-Adipogenic Effect of Theabrownin is Mediated by Bile Acid Alternative Synthesis Via Gut Microbiota Remodeling

Supplementary material

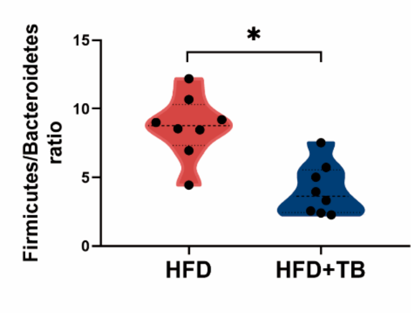

Supplementary Fig S1

Relative abundance of Firmicutes/Bacteroides as a ratio at the phylum level. (n=8 per group) . Differences between data were assessed using the Mann-Whitney U test. # p<0.01, ✱p<0.05

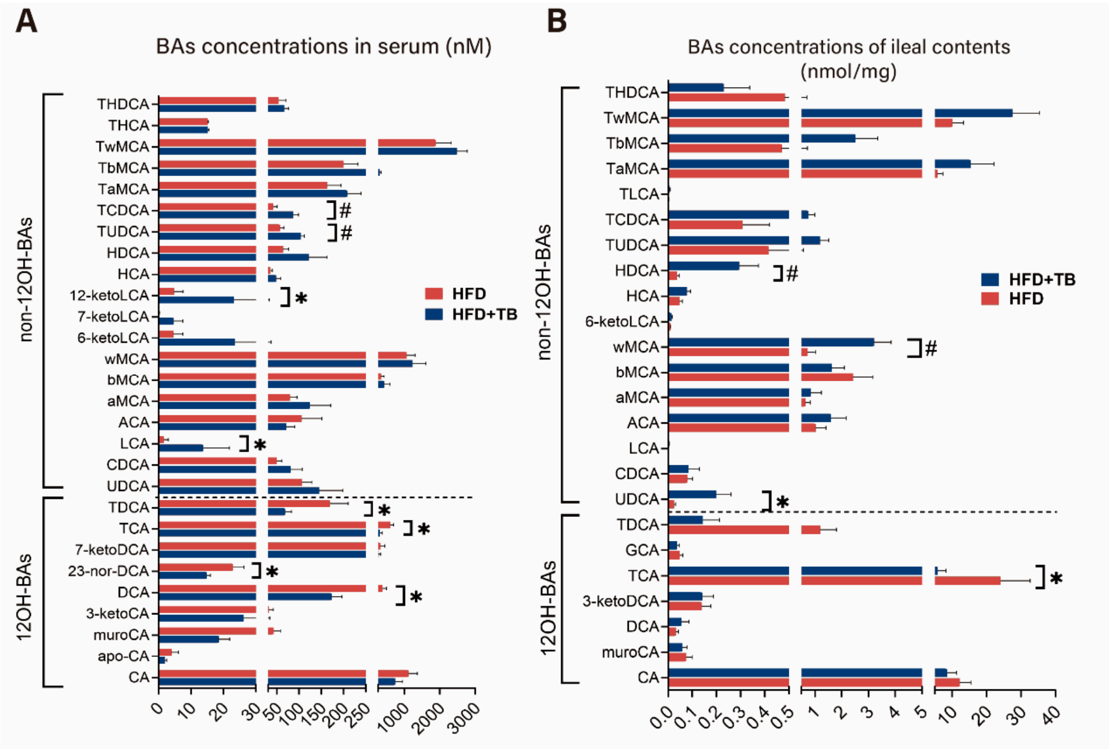

Supplementary Fig S2

(A) Altered BA profiles in serum induced by oral administration of theabrownin for 8 weeks in HFD-induced obesity mice(n=8 per group). (B) Altered BA profiles of ileal contents induced by oral administration of theabrownin for 8 weeks in HFD-induced obesity mice(n=8 per group). Differences between data were assessed using the Mann-Whitney U test with Benjamini-Hochberg adjustment. #p<0.01, ✱p<0.05

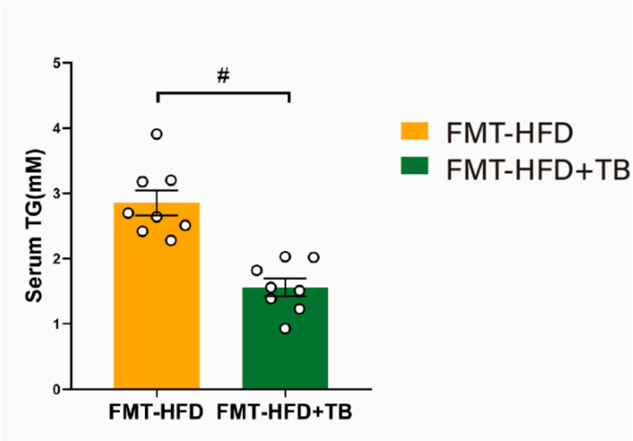

Supplementary Fig S3

TG level in the serum of germ-free mice transplanted with microbiota from control (FMT-HFD group) and theabrownin (FMT-HFD+TB group) treated mice (n=8 per group). Data are presented as the mean±SEM. Differences between data were assessed using the Mann-Whitney U test. #p<0.01, *p<0.05

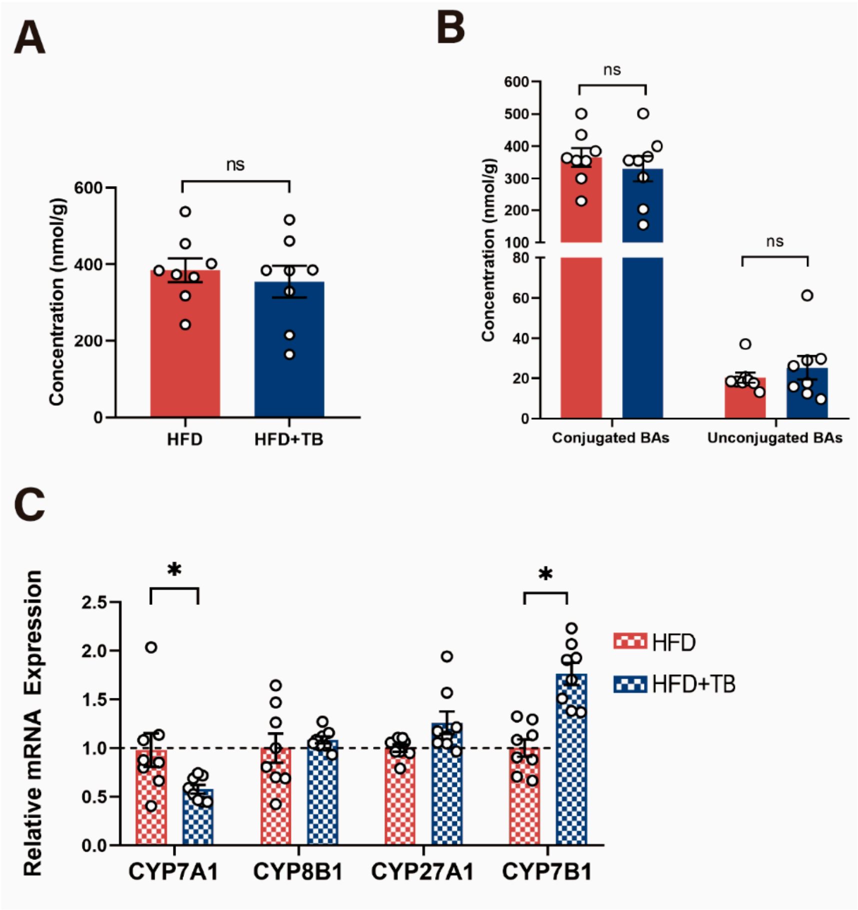

Supplementary Fig S4

(A) Concentrations of hepatic total bile acids (n=8 per group). (B) Concentrations of conjugated and unconjugated bile acids in the liver (n=8 per group). (C) mRNA

levels of Cyp7a1, Cyp8b1, Cyp27a1, and Cyp7b1 in the liver after theabrownin administration in HFD obese mice for 8 weeks. (n=8 per group). Data are presented as the mean±SEM. Differences between data were assessed using the Mann-Whitney U test. # $p<0.01$, * $p<0.05$
